# Patterns, Mechanisms and Genetics of Speciation in Reptiles and Amphibians

**DOI:** 10.3390/genes10090646

**Published:** 2019-08-26

**Authors:** Katharina C. Wollenberg Valero, Jonathon C. Marshall, Elizabeth Bastiaans, Adalgisa Caccone, Arley Camargo, Mariana Morando, Matthew L. Niemiller, Maciej Pabijan, Michael A. Russello, Barry Sinervo, Fernanda P. Werneck, Jack W. Sites, John J. Wiens, Sebastian Steinfartz

**Affiliations:** 1 Department of Biological and Marine Sciences, University of Hull, Cottingham Road, Hull HU6 7RX, UK; 2 Department of Zoology, Weber State University, 1415 Edvalson Street, Dept. 2505, Ogden, UT 84401, USA; 3 Department of Biology, State University of New York, College at Oneonta, Oneonta, NY 13820, USA; 4 Department of Ecology and Evolutionary Biology, Yale University, New Haven, CT 06520, USA; 5 Centro Universitario de Rivera, Universidad de la República, Ituzaingó 667, Rivera 40000, Uruguay; 6 Instituto Patagónico para el Estudio de los Ecosistemas Continentales (IPEEC, CENPAT-CONICET) Bv. Brown 2915, Puerto Madryn U9120ACD, Argentina; 7 Department of Biological Sciences, The University of Alabama in Huntsville, Huntsville, AL 35899, USA; 8 Department of Comparative Anatomy, Institute of Zoology and Biomedical Research, Jagiellonian University, ul. Gronostajowa 9, 30-387 Kraków, Poland; 9 Department of Biology, University of British Columbia, Okanagan Campus, 3247 University Way, Kelowna, BC V1V 1V7, Canada; 10 Department of Ecology and Evolutionary Biology, University of California, Santa Cruz, Coastal Biology Building, 130 McAllister Way, Santa Cruz, CA 95060, USA; 11 Programa de Coleções Científicas Biológicas, Coordenação de Biodiversidade, Instituto Nacional de Pesquisas da Amazônia, Manaus 69060-000, Brazil; 12 College of Life Sciences, Brigham Young University, Provo, UT 84602, USA; 13 Department of Ecology and Evolutionary Biology, University of Arizona, Tucson, AZ 85721, USA; 14 Molecular Evolution and Systematics of Animals, Institute of Biology, University of Leipzig, Talstrasse 33, 04103 Leipzig, Germany

**Keywords:** ecological speciation, niche, traits, taxonomy, genomics, phylogeography, phylogenetics, integrative taxonomy

## Abstract

In this contribution, the aspects of reptile and amphibian speciation that emerged from research performed over the past decade are reviewed. First, this study assesses how patterns and processes of speciation depend on knowing the taxonomy of the group in question, and discuss how integrative taxonomy has contributed to speciation research in these groups. This study then reviews the research on different aspects of speciation in reptiles and amphibians, including biogeography and climatic niches, ecological speciation, the relationship between speciation rates and phenotypic traits, and genetics and genomics. Further, several case studies of speciation in reptiles and amphibians that exemplify many of these themes are discussed. These include studies of integrative taxonomy and biogeography in South American lizards, ecological speciation in European salamanders, speciation and phenotypic evolution in frogs and lizards. The final case study combines genomics and biogeography in tortoises. The field of amphibian and reptile speciation research has steadily moved forward from the assessment of geographic and ecological aspects, to incorporating other dimensions of speciation, such as genetic mechanisms and evolutionary forces. A higher degree of integration among all these dimensions emerges as a goal for future research.

## 1. Synopsis

Reptiles and amphibians collectively span much of the tetrapod diversity. Living amphibians (~8000 species) form a monophyletic group, consisting of caecilians (~200 species), caudates (salamanders and newts; ~800 species), and anurans (frogs and toads; ~7000 species) [1]. Reptiles do not form a monophyletic group unless birds are included. They include the tuatara (1 species), squamates (lizards and snakes; ~10,000 species), turtles (~350 species), and crocodilians (24 species) [2]. Here, this study provides the first synthesis of research on speciation in amphibians and non-avian reptiles. While the body of amphibian and reptile speciation literature is too large to summarize in one contribution, this study gives snapshots of some of the most important speciation patterns and processes, and links these to case studies based on our own work and other recent developments in the field over the past decade.

Understanding speciation is a major goal of evolutionary biology. While numerous concepts of species exist, Mayr’s biological species concept of reproductively isolated populations [3] are adopted for simplicity. Speciation is defined as the origin of barriers to reproductive isolation [4]. Speciation can arise as a consequence of divergent selection (extrinsic factors) or through genome properties such as genomic conflict (as intrinsic factors) [4]. Considerable progress towards understanding the processes of speciation can be made by focusing on particular groups of organisms (e.g., birds [5]). The accurate assessment of patterns and processes of speciation primarily depends on a good knowledge of taxonomy and systematics of the group in question [6]. This study first discusses how integrative taxonomy has helped to clarify operational taxonomic units that have then been used to infer evolutionary processes in reptiles and amphibians (Section 2.1 and Section 3.1). Speciation in amphibians and reptiles is thought to be influenced by numerous factors, which can be categorized as extrinsic and intrinsic. Extrinsic factors represent the overall opportunity for speciation provided by the environment, while intrinsic factors represent the specific organismal potential to diversify, in relation to its existing evolutionary constraints [7]. The importance of extrinsic factors is evident from patterns of species richness. Amphibian species richness is concentrated in regions with high net primary productivity [8], while reptile species diversity on a global scale is correlated with temperature and topography in the Afrotropics [9,10]. The importance of such bioclimatic niches, and bioclimatic clines on amphibian and reptile speciation both generally and in form of specific examples are discussed (Section 2.2, Section 3.2 and Section 3.5). Intrinsic factors, in contrast, include ecological specialization [11,12,13] (Section 2.3 and Section 3.3), ecologically relevant traits such as body size or coloration (Section 2.4 and Section 3.4), metabolic rate [14], population density [15,16,17], structural chromosome rearrangements [18,19,20,21,22,23], or parameters related to reproduction [24,25,26] (Section 3.3). To obtain a quantitative understanding of the process of diversification in clades of reptiles and amphibians, the interplay of both extrinsic and intrinsic factors needs to be assessed. For example, Tilley, Verrell and Arnold [27] compared biogeographic patterns to levels of ethological isolation in a plethodontid salamander species [28], whereas other studies correlated phylogeographic patterns with the evolution of other traits [29,30,31] or tested for the degree of speciation as related to reproductive isolation in certain groups [32]. The interplay between extrinsic and intrinsic factors in shaping species distributions, and the patterns of endemism and species richness [33], is also evident in the parameter of geographic range size [34]. The range size of a species is both linked to environmentally suitable niches, and to intrinsic factors limiting dispersal, such as body size (Section 3.4). With regards to the possible mode of speciation, timing also seems to be important. Intriguingly, the present species diversity of some island radiations is an outcome of speciation events that post-date the initial burst of divergence events in the early stages of the radiation [35,36]. Intrinsic factors (Section 2.5) may help to explain more recent speciation events in adaptive radiations that often do not coincide with phylogeographic splits associated with hard dispersal barriers. Lineages can furthermore diverge across environmental clines in the presence of gene flow (Section 3.5). Alternatively, the signatures of extrinsic factors could be more likely to prevail over long time spans, while some intrinsic processes might not necessarily result in speciation, and translate into data that are more difficult to interpret. Many systems for studying the process of speciation look at lineages that are divergent, but do not show signatures of complete reproductive isolation, such as the classic example of the ring species complex of *Ensatina eschscholtzii* salamanders in California [37]. Since speciation cannot be studied anymore once the process is complete, criticisms of this practice have centered on the fact that such lineage divergence or incipient speciation [38,39] may not eventually result in speciation as the complete speciation process would be more readily observed in laboratory [40] or semi-natural settings [41]. This approach, however, has its own downsides, and has not been performed on amphibians or reptiles yet. Section 3.3 outlines how present lineage divergence can still be used to better understand population genetic mechanisms important for speciation [42], while in Section 3.6, genomic properties in diverging lineages and related fusion/fission dynamics including de-speciation (the secondary collapse of an emerging reproductive barrier) are discussed.

## 2. Aspects of Amphibian and Reptile Speciation

### 2.1. Integrative Taxonomy Builds the Foundation of Modern Speciation Research

Speciation research in reptiles and amphibians has been facilitated by progress in integrative taxonomy (IT) over the past decade [43,44,45], which has provided increased statistical rigor for species delimitation [46], and has aided in a better understanding of historical biogeography [47]. IT combines different kinds of data and methods for species discovery [48,49,50,51] and includes step-by-step methods based on sequential analyses of independent data types, followed by a qualitative assessment of species boundaries [52,53]. IT approaches can also use model-based methods that simultaneously evaluate multiple data types, with subsequent delimitation of species based on statistical or information criteria [54,55,56]. The four focal areas of IT are: (a) The validation of candidate species as evolutionary distinct lineages; (b) inferring species relationships; (c) detecting cryptic diversity; (d) the assignment of individual specimens to a species group [54,57]. The dense geographic sampling and mtDNA sequencing can be utilized as a first pass approach for poorly known groups. The species hypothesized from this first approach (candidate species [6]) can be used to direct further sampling. IT can then be used to test species limits (i.e., including other molecular markers, combined with data on morphology and ecology).

This approach is useful for example, when divergence initially occurs along non-molecular axes of differentiation, and/or when divergence occurs with gene flow, as is the case in the South American lizards *Liolaemus bibroni* and *Lacerta gracilis* [58]. Section 3.1 outlines in detail how integrative taxonomy methods have been applied to South American liolaemid lizards and has helped to improve biogeographic hypotheses (Section 3.2). The recent availability of genomic data has led to a deeper understanding of the genomic basis of traits, and genome-level processes during speciation [59,60]. The processes, such as reticulate evolution, are becoming better known as an important aspect of speciation with the availability of these data sets [61,62]. It is becoming evident that their analysis requires methods (such as network-based approaches) that go beyond those based on bifurcating trees. For assessing instances of incomplete speciation events, a number of recent methods based on the multispecies coalescent network are now available (e.g., PhyloNetworks [63]; PhyloNet [64]; SpeciesNetwork in BEAST2 [65]). However, so far, these methods are only able to handle a limited number of taxa when using genome-wide data.

Recently, model-based species delimitation has begun to incorporate the use of artificial intelligence-based methods [66,67] to identify or predict species. The species-identifying artificial intelligence (SIAI) has been used to identify species of plankton via microscopic images and bat species via their calls [68,69]. In frogs, the concept has similarly found application through the use of AI-based classification using bio-acoustic monitoring data [70,71]. The authors suspect that in the near future, the methods are expected to become available that can extend AI-based classification to identifying novel species by focusing on the description of unclassified samples. However, despite the appeal of such methods to non-specialists and their apparent ease of use, image-based species description has received substantial criticism from experts because of problems aligned with the fluid definition of diagnostic criteria over time, and the associated need for the preservation of type specimens [72]. Additionally, the lack of understanding of the black box nature of some neural network algorithms used in machine learning for such work implies that caution is needed when using these methods for management or conservation predictions [73].

Independently from species delimitation methods, the field of herpetology recently has undergone a period of enthusiastic lineage splitting [74,75]. Hillis [74] recently provided a perspective on species delimitation in herpetology, arguing that taxonomic classifications should be viewed primarily as a service from experts to non-experts. Consequently, they should facilitate, not complicate, the use of binomials as operational units of further analysis.

### 2.2. The Importance of Biogeography and the Climatic Niche

The niche may play many pivotal roles in speciation. The niche describes the set of abiotic and biotic conditions in which a species can persist [76,77], including both the environmental conditions that determine their broad-scale distribution (Grinnellian niche) and their interactions with other species at the local scale (Eltonian niche). The niche is critical to speciation in at least two ways. First, the Grinnellian niche plays an important role in geographic isolation. This is clear from the first principles, given that the niche determines where species occur. Both divergence and conservatism in the niche may play a critical role in speciation.

For parapatric speciation, niche divergence of adjacent populations along an ecological gradient may lead to some populations becoming locally adapted to different parts of the gradient [78,79]. This may then lead to reduced gene flow between these populations, possibly leading to parapatric speciation (e.g., if individuals of one population cannot tolerate the local environmental conditions where the other population occurs, and vice versa). This scenario typifies the process of speciation via niche divergence. One classic scenario for parapatric speciation through niche divergence involves different climates along a mountain slope (Section 3.5).

For allopatric speciation, niche conservatism may lead to the initial geographic isolation of populations [79,80]. Niche conservatism is the tendency of species to retain niche-related ecological traits over time [81]. From the first principles, niche conservatism should be critical for allopatry [80]. Populations become allopatric when they are separated by a barrier of unsuitable ecological conditions. This barrier may be relatively obvious (e.g., oceans for terrestrial species) or more subtle (lowland mesic temperate forest versus upland mesic temperate forest), but the basic principle is the same. Ultimately, the reason why this barrier functions as a barrier is that the populations separated by the barrier are unable to adapt to the ecological conditions within that area and maintain gene flow across it. Thus, the barrier of unsuitable ecological conditions is maintained by the retention of similar niche-related ecological traits in these populations over time (i.e., niche conservatism). It is very important to note however, that just because niche conservatism was involved in the initial geographic isolation of the populations, this does not mean that they do not diverge subsequently in one or more ecological traits.

There are now many examples in the literature of speciation through both niche divergence and niche conservatism in reptiles and amphibians, especially for the climatic niche. For example, there is evidence that in tropical salamanders, sister species tend to occur in divergent climatic conditions [82]. At a larger scale, tropical plethodontid clades with higher rates of climatic niche evolution have faster rates of diversification (speciation minus extinction), consistent with the idea that climatic divergence drives speciation [83]. Climatic niche widths for temperature-related variables appear to be narrower in the tropics [84,85], including in reptiles and amphibians [86], but whether this increases climatic niche divergence and speciation remains unclear [82,87]. At an even broader phylogenetic scale, the levels of climatic niche divergence seem to explain much of the variation in the diversification rates among salamander and frog families, with greater climatic niche divergence within families associated with higher rates of diversification [88]. Indeed, climatic niche divergence is far better at predicting family-level diversification rates than climatic niche variables alone (i.e., tropical versus temperate). Similar patterns have been found using rates of climatic-niche divergence in frogs [89]. Several other studies have found divergent climatic niches between closely related species, including studies of frogs [90], lizards [91], and snakes [92]. Several studies have also found interesting patterns of current within-species phenotypic divergence and environmental variation that eventually may lead to parapatric speciation [93,94,95]. Several other studies potentially support speciation through climatic niche conservatism, including analyses of plethodontid salamanders in eastern North America [96,97], Australian frogs [98], and the studies of various groups of tropical terrestrial vertebrates [87]. A survey of 49 allopatric species pairs in squamates suggests that climatic niche divergence drove speciation in ~70% and climatic niche conservatism drove speciation in ~20% [99]. It is also important to note that just because climatic niche conservatism was not supported as driving allopatric speciation, allopatry may have been associated with niche conservatism in other ecological traits (e.g., microhabitat types, such as rocks or sand).

The Eltonian niche may also be important in speciation. For example, many models of adaptive radiation suggest that an important part of the process involves divergence along many different axes of the ecological niche, including axes that involve division of resources at the local scale. For example, many vertebrate radiations involve divergence in microhabitat and body size, suggesting that these are linked to diversification [100] (Section 3.4). However, directly linking variation in some of these traits to speciation (or diversification) has proven difficult [101]. Nevertheless, the microhabitat (aquatic versus terrestrial) seems to explain the majority of the variation in the diversification rates (~67%) among the 12 major clades of vertebrates [102]. A microhabitat is also an important predictor of the diversification rates across frog families [89] and squamate families [103], with predominantly arboreal clades showing higher rates (in both clades) and aquatic and fossorial lineages showing lower rates (in squamates; for similar results in snakes see also [104]. Another important question is whether speciation along Eltonian niche axes might reflect sympatric speciation (within the same geographic area).

Clearly, the role of the niche in speciation depends (in some part) on the geographic mode of the speciation involved. The question arises about what is known about geographic modes of speciation in reptiles and amphibians. In general, allopatric speciation has widely been considered the most common geographic mode [105]. Several herpetological studies now show some support for this hypothesis. For example, the studies of the range overlap of species pairs in some groups support the prevalence of the allopatric mode (salamanders [96]; frogs [90]; turtles [106]). Among 242 sister species pairs of squamates surveyed [98], allopatric pairs are most common (41.3%), but other geographic patterns are also common, including many parapatric (19.4%), partially sympatric (17.7%), and fully sympatric pairs (21.5%). However, other groups remain largely unsurveyed in terms of their geographic modes and the possibility of the post-speciation range shifts needs to be considered.

### 2.3. Ecological Speciation

The adaptation of individuals to new or differing environmental conditions can cause the adaptive divergence of populations leading to speciation, if natural selection strongly favors different ecotypes and reproductive isolation evolves as a consequence of such a differential habitat use [107,108,109,110]. This ecological or adaptive speciation has been identified as a major biological process that has shaped species diversity in quite distinct taxa, including Darwin’s finches, three-spined sticklebacks, pea aphids, and *Rhagoletis* flies [109]. Another example are *Anolis* lizards occurring on the islands of the Lesser Antilles, where transect sampling efforts along environmental gradients have enabled the identification of both historical population effects, and ecological effects. The populations that have diversified in allopatry showed less reproductive isolation amongst each other, than populations that diversified across habitat gradients [94,111,112]. Ecological speciation is also considered a major process underlying adaptive radiations, which describes the process of rapid and frequent speciation from a common ancestor [113]. During the well-studied adaptive radiation of Darwin’s finches across the Galapagos archipelago, for example, 14 distinct species and subspecies have formed starting from a single colonization event from the South American mainland, roughly 1.6 million years ago [114]. However, the process of ecological speciation can also occur within shorter periods. In three-spined stickleback, repeated and parallel lineage divergence of limnetic and benthic forms inhabiting small lakes in southern British Columbia followed the last glaciation only a few thousand years ago [115,116].

Many species showing population subdivision and genetic divergence linked to habitat adaptation are not characterized by complete reproductive isolation. However, they may reflect different stages of adaptive divergence along a continuum reaching from pure adaptive-ecological variation without reproductive isolation, to ecological-adaptive differences associated with irreversible reproductive isolation (e.g., in fish [109]). As proposed by Tautz [117], adaptive or ecological speciation follows distinct phases through time associated with the change in adaptive traits and neutral genetic divergence arising from speciation (see Figure 15.1 in Tautz [117]). Initially, in phase 1, individuals use or exploit different environmental niches and traits that allow them to use different resources, and diversify quickly into different ecotypes associated with different resources. Assuming a two-ecotype scenario, individuals should mate assortatively with their own ecotype to avoid producing sub-optimally adapted offspring when interbreeding with the other ecotypes. At this early stage of adaptive speciation, neutral genetic divergence between the gene pools of corresponding ecotypes is not necessarily observable (i.e., at this stage, ecotypes should not show signs of genetic divergence as measured by neutrally evolving loci across the genome). However, the genes underlying adaptive traits (e.g., beak shape and size in Darwin’s finches for example [118]) should show signs of selection and may differ in allele frequencies, the degree of polymorphism, etc. In phase 2, the differentiation of adaptive traits becomes more pronounced and gene pools of ecotypes should show signs of neutral genetic divergence. At this phase, genetically differentiated subpopulations can be observed. During phase 3, no further differentiation of adaptive traits can be observed. However, genetic differentiation is expected to increase further, given the reproductive isolation of ecotypes. Following this phase, it is difficult to predict how adaptive traits will evolve, but ecotypes have evolved into phylogenetically distinct species showing strong neutral divergence. Importantly, many natural systems that have been studied for ecological speciation have not reached the final stage of complete speciation (e.g., some cichlid fishes [119]). Nevertheless, these represent exciting study systems that may show how ecological adaptation can cause genomic divergence via selection [42] and potentially affect the population structure over time.

The processes and mechanisms of ecological speciation can be best studied in situations where the direct impact of ecological adaptation is measurable with genetic markers. One example is when habitat difference metrics are correlated with genetic differences. Furthermore, it is also useful to find situations in which spatial impacts, such as geographic isolation, can be ruled out as primary factors causing genetic differentiation. Therefore, the individuals or populations under investigation should ideally be in spatial contact. Section 3.3 describes another exciting study system for ecological speciation, the European Fire Salamander (*Salamandra salamandra*). Here, the adaptation of salamander larvae to different habitat types has caused adaptive divergence within a salamander population, with consequences for population structure and behavior. This system may represent an early stage of ecological speciation.

### 2.4. Speciation Rates and Variable Traits

Even the most superficial look at the Tree of Life immediately reveals enormous differences in species diversity among clades [102,120,121]. Some taxa such as extant coelacanths, the tuatara, the platypus or the two pig nose frogs (*Nasikabatrachus* spp.) are the lone representatives of ancient lineages and are sometimes referred to as living fossils. On the other hand, other clades of comparable age may contain thousands of species. The diversification rates are composed of speciation and extinction rates, and it is usually not easy to disentangle these two factors. In amphibians, rapidly speciating clades are also more threatened by extinction [122]. The species-poor extant clades might have been much more diverse in the past, and suffered from high extinction rates. In contrast, most species-rich extant clades are explained by high diversification rates [123], but the reasons underlying these differences in speciation rates remain unknown. Butlin and colleagues [124] flagged this as one important unsolved question in speciation research. The most frequent approach to the study of speciation or diversification rates and their possible determinants is to use phylogenies and comparative methods, but these methods require refinement to be able to distinguish between the effects of speciation and extinction [124,125,126].

Speciation rates, as well as species diversification rates and population divergence, can also be influenced by phenotypic traits [127]. The diversification rates across all animals were not impacted by body size [127]. The connection between body size and speciation rate is difficult to disentangle in smaller groups (e.g., teleost fish, [128]). Some large adaptive radiations and species-rich clades of mammals and lizards are comprised of small-bodied species [129], but this does not necessarily mean that body size drives rapid diversification in these groups. The rates of change in the body and the shape size are unrelated to the diversification rates in plethodontid salamanders [101]. Section 3.4 outlines how body size shaped the adaptive radiation of Madagascan and other frogs. The intrinsic factors, such as organismal traits, that enable the colonization of new environments, or the more abstract concept of ecospace [130], are referred to as key innovations. These key innovations are thought to influence the diversification rates. Ecospaces recurrently occupied by amphibian clades are arboreal versus terrestrial versus aquatic, and terrestrial (endotrophic) reproduction including viviparity. The morphological and physiological traits which allow these switches are largely unstudied. Arboreality (but not other microhabitats [89]) has been identified to increase the diversification rates in frogs, which constitutes an interesting avenue for future study. The life history mode was found to be unrelated to the diversification rates across frogs [131]. Terrestriality did not increase the diversification rate in the frog genus *Phrynobatrachus*, in which the more terrestrial clades showed decreased rates [132]. The presence of aerolate ventral skin was found to be correlated with increased species richness in South American *Terrarana* frogs [133]. One interpretation of this latter finding is that more vascularized bellies may have been an adaptation to lower atmospheric oxygen levels, facilitating the colonization of high-altitude ranges. In bufonids, a suite of morphological and life history traits have been demonstrated to increase the colonization ability and trigger diversification [134]. This range expansion phenotype includes a terrestrial niche, large body size, the presence of parotid glands and inguinal fat bodies, aquatic oviposition sites, large clutch size and exotrophic larvae.

Apart from the body size, many amphibians and reptiles have bright colors. When these colors vary within and among populations, they are called color polymorphisms. If such polymorphic lineages are less vulnerable to extinction, they may also be more diverse than monomorphic lineages and tend to be older, as is the case of snakes [135]. Alternatively, the older clades may simply accumulate polymorphic loci over longer periods so that it is not trivial to disentangle cause and effect. The presence of multiple morphs may allow populations to occupy more than one ecological niche and/or maintain higher levels of genetic diversity than are present in monomorphic populations [136,137,138]. However, Bolton, Rollins and Griffith [139] suggest that some features of color polymorphic populations may make them more vulnerable to extinction than monomorphic populations. Both theoretical [136,137,140,141] and empirical [138,142,143] studies support the idea that taxa in which color polymorphisms or alternative reproductive strategies are common may exhibit higher rates of speciation than taxa in which most populations are monomorphic for these traits. Besides color in the human visual spectrum that generate color morphs of Phrynosomatidae and Lacertidae, UV-coloration appears to be important in speciation of green lizard in two lineages that come into contact, *Lacerta viridis* and *L. bilineatus* [144] and generate hybrid unfitness [145,146].

Sexual selection acting upon color polymorphisms is an important driver for population divergence to evolve, and thus important for understanding the early stages of speciation [140,147].

In squamate reptiles, especially lizards, the populations of many species include two or more discrete color morphs within one or both sexes. In most cases where the proximate basis of such color variation has been studied, morphs are highly heritable [148,149,150]. A key insight into the mechanisms governing color morphs of all species of lizards includes genome studies of the potential genetic factors controlling morphs, exemplified by a recent paper by Andrade and colleagues [151] that shows both pteridines and carotenoid genes control the color of *Podarcis muralis*. Pteridine expression and carotenoids have also been linked to the control of yellow and orange color morphs in the side-blotched lizard using biochemical studies, but that the blue color morph arises from iridophore reflecting platelets [152]. This finding is supported by studies on the trimorphic lacertid *Zootoca vivipara* that show iridophores control color [153]. Combined, these genomic, and biochemical studies suggest a multi-component signal to the mating systems of males with three color morphs and thus, a more complex etiology than a simple one locus gene.

In addition to their differences in color, morphs differ in one or more aspects of reproductive behavior in numerous species, including members of the families Phrynosomatidae [154,155,156,157], Lacertidae [158,159,160], and Agamidae [161,162]. The males of different color morphs may vary in aggression, dispersal, physiological performance, territoriality, and/or mate choice [150,154,156,159,161,163,164,165,166]. The female morphs may differ in life history, maternal effects, and/or mate choice [158,167,168,169,170,171,172,173,174]. Within the populations, heritable color and behavioral morphs may be maintained by negative frequency-dependent selection, temporally or spatially variable selection, overdominance, or gene flow between the populations differing in coloration [142,175]. However, while mate-choice based selection on polymorphisms might drive population divergence, environmental or ecological factors are very strong drivers for speciation compared to sexual selection [141] or phylogeographic structure [176,177,178], an idea that is supported by recent findings in snakes and lizards. In numerous color-polymorphic taxa, closely related species [179,180] or populations of the same species [181,182,183] vary in the number or frequency of morphs present [184]. In the well-studied side-blotched lizard, *Uta stansburiana*, the collapse of trimorphic rock-paper-scissors mating systems to di- and monomorphic states is driven by the interaction of morph fitness in warm versus cool climates, implying a strong interaction between the social system and ecophysiology [185]. After morph loss [183], other reproductive and sexually selected traits rapidly evolve to new equilibria [186]. These patterns imply that an interaction between ecological and social factors drives the evolution of new ecotypes, which can promote reproductive isolation between the populations that differ in morph numbers [143]. It is important to note that morph-frequency variation might alternatively occur due to stochastic processes, such as genetic drift or founder effects [179,187,188]. As Butlin and colleagues [124] pointed out, reproductive isolation is still one of the best criteria upon which to assess any factors putatively contributing to speciation. The presence of different morphs in closely related populations may contribute to prezygotic [189] or postzygotic reproductive isolation [143] between those populations. Further species-wide studies comparing rates of gene flow between the populations differing in morph frequencies would be helpful in empirically evaluating the effect of polymorphism on reproductive isolation between the populations. One recent example of such work found that in the lizard *Ctenophorus decresii*, only limited gene flow occurred after secondary contact between polymorphic and monomorphic lineages [188].

In frogs, the variation in male advertisement calls (calls hereafter) has long been considered a key trait that potentially drives their speciation. However, studies that have definitively shown this remain rare to date. It is clear that different species of frogs have different calls. Furthermore, there are examples where female frogs seem to prefer conspecific calls over heterospecific calls (e.g., in *Physalaemus* frogs; [190]. One of the best-case studies of potential call-driven speciation involves different populations of *Physalaemus petersi* in the western Amazon Basin in South America [191]. In this system, some populations differ in their call types (complex versus simple), and these differences have evolved repeatedly and become fixed more quickly than expected by drift. The females generally prefer the males with calls of their native population. Furthermore, there is strongly restricted gene flow between adjacent populations with different call types. There is also evidence for speciation driven by reinforcement on the call variation in Australian treefrogs (*Litoria*; [26]). Other important systems in which calls are important to reproductive isolation include North American spadefoot toads (*Spea*; [192,193]) and chorus frogs (*Pseudacris*; [194,195]). An unresolved challenge for studies of frog speciation is to determine whether call variation is the initial cause of lineage splitting or merely helps distinct lineages remain distinct (especially given that many frog species appear to arise in allopatry, where call differences are expected to be irrelevant to speciation). Interestingly, an important cause of reproductive isolation among populations in several systems may be call divergence between conspecific populations where some populations are sympatric with heterospecifics ([26,193,195]).

### 2.5. Genome Properties and Processes

Pure allopatric speciation has long been thought to be the prevalent mechanism of speciation [196], and the evidence outlined above shows that it is also very common in amphibians and reptiles. However, the alternative hypothesis (not purely allopatric speciation) is harder to test, as species with an allopatric distribution lend themselves to inferring past allopatric speciation from it, whereas speciation mechanisms in species with overlapping distribution areas and that might involve some amount of the gene flow are harder to infer. Allopatric speciation has often been inferred across hard barriers to the gene flow, which are thought to limit the gene flow completely. Soft barriers to the gene flow limit dispersal but still allow for low levels of migration [197]. This may lead to the existence of metapopulations with more or less continuous distribution, which can be deeply divergent across the area. These scenarios are harder to interpret in terms of speciation processes. The question arises whether the standing local adaptive variation, where local variants have evolved and are maintained despite a low number of migrants departing and arriving, eventually result in complete reproductive isolation. Some very young sympatric or even syntopic and microendemic Madagascan sister pairs of frogs have been studied within the context of this question. At the phenotype level, recently diverged species living in syntopy can show evidence for ecological speciation coinciding with soft barriers to the gene flow. These include divergence in bioacoustics characters (*Gephyromantis eiselti* and *Gephyromantis thelenae*, [198]), divergence in body size (*Gephyromantis enki* and *G. boulengeri* [199]), or functional convergence in color patterns (for a case of mimicry, cf. *Mantella madagascariensis* and *Mantella baroni* [200]).

On small spatial and temporal scales, incomplete speciation on an evolutionary trajectory to be completed could be distinguished from a stable metapopulation scenario by a combination of the following lines of evidence: (1) Assuming that similar processes of selection result in similar outcomes, the divergence patterns can be compared across different taxonomic levels of one clade inhabiting similar environments. For example, if speciation processes were deterministic, then similarities in the patterns of character divergence among diverging populations and among young species of the same clade are expected to be found, which would indicate that the populations are on a similar trajectory to diversify. Comparing different clades occupying the same habitat (e.g., different endemic radiations of Madagascar showing similar phylogeographic patterns) allows inferring common evolutionary processes among them [201]. (2) If signatures of convergent genomic adaptation among several populations can be found, this may indicate adaptive speciation to a common set of environmental variables that promote speciation [202]. The beneficial convergent alleles can either evolve thorough independent mutational events, or through selection on a polymorphism in the common ancestor. Further, it is likely that more than one genomic route can produce a phenotypic adaptation responsible for divergence with the gene flow, as genomic adaptations in different loci may be functionally equivalent (polygenic) and cause similar phenotypes (homoplasy). For example, several mutations can cause interruption of the same metabolic pathway at different levels [203].

The existence of divergence and speciation in the presence of the gene flow has been proven in recent years by studying divergence at the genomic level [204,205,206,207,208,209]. It is widely accepted that speciation requires the interruption of the gene flow between populations [105], as gene exchange and recombination is a significant impediment to population divergence and the formation of new species. Speciation among allopatric populations is generally straightforward, as typically an ecological barrier to the gene flow can be identified (Section 3.3). In the absence of such a barrier, another mechanism was required to counterbalance the homogenizing effect of the gene flow. Theoretical models have demonstrated a variety of scenarios in which speciation can occur without complete geographic isolation [210,211,212,213], and empirical examples demonstrate that speciation in the face of the gene flow may be more common than previously thought [110,209,214,215]. In the early stage of speciation, the extent of the gene flow can be estimated via the shape of the distribution of the genome-wide Fst statistic. A more binomial shape of this distribution indicates a lower number of driver loci involved in divergence with the gene flow, while a wider curve indicates that divergence with less gene flow proceeds gradually across many loci [4,216]. Several processes can facilitate genetic divergence during speciation in the face of the gene flow. Among these are direct divergent selection on a few loci of large effects that underlie reproductive isolation [209], mate choice that is correlated with a trait under divergent selection [204,210], and divergence hitchhiking in which gene exchange is reduced over larger genomic regions as an indirect effect of strong divergent selection on loci involved in local adaptation [217,218]. More recently, advances in genomic approaches have extended the study of speciation with gene flow by examining patterns and extent of admixture, divergence, and linkage disequilibrium between taxa on a genome-wide scale [209,215,219,220]. Amphibians and reptiles have been important model systems in recent years in determining the prevalence, patterns, processes, and mechanisms of divergence and speciation with the gene flow. Examples of studies that have examined aspects of divergence and speciation with gene flow include tropical frogs [90], Andean frogs [221], ranid frogs [222,223,224,225], barking frogs [226], chorus frogs [227], newts [228,229], plethodontid salamanders [82,230], *Anolis* lizards [94,231,232], Iberian and North African wall lizards [233], *Sceloporus* spiny lizards [234,235,236,237,238], whiptail lizards [239], rattlesnakes [240], *Pantherophis* ratsnakes [241], and *Thamnophis* garter snakes [242,243]. A review of divergence with gene flow in amphibians emphasized the importance of genome-scale sequencing to understand gene-level versus genome-level processes in speciation [244]. However, only a few studies have begun examining speciation with gene flow using genomic data in amphibians and reptiles [226,236,240]. This might reflect the relatively higher cost of performing such studies, which is more accessible to well-funded labs. Section 3.5 discusses convergent phenotypic and genomic adaptations under incomplete lineage divergence in a species of *Anolis* lizard on the Caribbean island of Hispaniola.

A less explored aspect of speciation is, when it occurs without generating two bifurcating lineages. Reticulate evolution describes the emergence of new species-level lineages after a process of splitting and merging of population-level lineages, which can occur in both sympatry and allopatry. In the absence of complete isolation of the populations, (e.g., when populations diverge through ecological factors), this reticulated nature of divergence is not only represented by merging and splitting populations. It may also be represented by splitting and merging of different parts of the genome. The different portions of the genome that are more or less related to the selection pressure may diverge at different speeds among populations [39,216,245]. In such a scenario, phylogenies produced by different genes might yield different topologies (see also Section 3.5).

One related nonlinear speciation process based on intrinsic reproductive isolation is introgressive hybridization where genes from different lineages are merging into the genome of another lineage. At its extreme, introgressive hybridization can result in the takeover of another lineage’s genome, which results in extinction by de-speciation [246]. For example, a study detected 5–10% of hybrids between the rare Florida bog frog *Lithobates okaloosae* and its more common congener (the green frog, *L. calamitans*) [247].

In comparison, true hybrid speciation involves the merging of entire genomes. As an outcome of this process, various types of clonal reproduction occur in a few species of frogs (hybridogenesis in European water frogs, both males and females of *Pelophylax*) and reptiles. In reptiles, this involves various types of facultative or true parthenogenesis, whereby offspring are only produced through participation of the female genome, which is therefore not recombined. Genetic variation, which may post-date the time of the initial hybridogenesis event, was however found in most investigated species. The lacertid *Darevskia (Lacerta) rostombekowi* had until recently been thought to represent a monoclonal lineage with no variation in allozyme markers [248]. However, a study using microsatellite loci has recently shown that post-formation genomic variation also exists in this species [249]. Parthenogenetic species can also originate without hybridization, as in the xantusiid lizard genus *Lepidophyma* [250].

It has recently become apparent that noncoding genomic elements such as transposons may also constitute an important intrinsic factor for speciation. Transposable elements (TEs) make up a large portion of the genome of the strawberry poison frog, *Oophaga pumilio* [251]. TE activity can rapidly cause genomic incompatibility and thus may serve as an intrinsic factor for reproductive isolation [252]. The random relocation of these elements during meiosis may promote speciation, given that bursts of transposable element (TE) activity (hot genomes) align with bursts of speciation in mammals [253] and *Anolis* lizards [254].

The early speciation stages sometimes are perhaps better represented through complex networks of fusions and fissions over time [255]. Even in some textbook examples for allopatric speciation, such as speciation in Galapagos giant tortoises across the islands of the archipelago [256], such introgressive hybridization fusion/fission dynamics have recently been identified as an important factor for their speciation history [257]. Through their relatively unique life history traits, Galapagos giant tortoises may represent an ideal model system to understanding such evolutionary dynamics at the early stages of speciation [258], which is discussed in Section 3.6.

## 3. Case Studies

### 3.1. Liolaemid Lizards—From Poorly Known Taxonomic Groups to Evolutionary Radiations

Bad taxonomy due to limited data, or inappropriate methods to assess species boundaries can lead to taxonomic inflation (the unnecessary assignment of nomina to lineages with only shallow divergence), which may lead to errors in estimating long-term diversification trends (i.e., accelerated speciation, low extinction rates, frequent ecological speciation). The lizard genus *Liolaemus* seems extremely species-rich, and has a long history of scientific investigation [259,260]. The genus ranges across a large part of southern South America, including Patagonian steppes and heterogeneous mountain landscapes that have been subjected to a variety of climatic and geological changes since the origin of the group (~55 million years ago [261]). The key questions about actual species diversity of the genus, and the processes that have generated and maintained this diversity, have been addressed in a number of recent studies with a combination of new molecular data, integrative taxonomic approaches, and modern methods of species delimitation and diversification.

*Liolaemus* is a large genus (~260 species) distributed from Tierra del Fuego to north-central Peru, from sea level to ~5000 m in elevation [259,262,263]. In 2003, there were ~160 described species, but after the first detailed molecular study of one species complex (*L. elongatus* complex), Morando, Avila and Sites [264] inferred that *Liolaemus* could contain at least twice the number of known species, based on the discovery of multiple well-supported mtDNA haploclades within this single taxonomic complex. Since 2003, 100 *Liolaemus* species have been described (based on morphology and on molecular data for many). Another ~55 have been identified as candidate species (based mainly on mtDNA and allopatry) that require further study. These numbers, added to the 160 known species from 13 years ago, brings the total number to 315 potential species. However, key geographic regions still remain poorly studied, and may contain many additional species. For example, portions of central and southern Patagonia and the sub-Antarctic provinces have barely been sampled for lizards in general [265,266].

During the last decade, many cryptic, candidate species within *Liolaemus* have been revealed by the application of dense geographic sampling, multiple DNA loci, coalescent-based and heuristic species delimitation methods. These species were supported by integrative taxonomic (IT) approaches comparing the divergence patterns of genetic, morphological (meristic and traditional/geometric morphometrics), and bioclimatic data. For instance, several candidate species were confirmed within the *L. elongatus* and *L. kriegi* complexes using multi-locus genetic data [267,268]. Based on the integration of molecular, morphological, and ecological niche envelope data, several new *Liolaemus* species were described from Perú [269]. Minoli and colleagues [270] tested species limits in the *Liolaemus fitzingerii* group with morphometric and niche envelope analyses, and a similar integrative taxonomic approach discovered new candidate species in the *L. lineomaculatus* section [271,272]. Aguilar and colleagues [56] recently applied an IT approach [53,54] to resolve some taxonomic uncertainties in the northernmost species of *Liolaemus*, the *montanus* group in north-central Peru. The results of this study revealed that as a rule, older candidate species, as identified by longer branches on the gene and species trees, were generally more clearly corroborated by other classes of data and across methods [56].

The hidden diversity within several clades of *Liolaemus* has led to further studies to investigate the evolutionary processes underlying these diversification patterns. Olave et al. [273] combined multiple loci and morphological data to resolve species boundaries in the *L. rothi* complex. They discovered strong genetic differentiation but limited morphological divergence, suggesting that selective pressures have produced phenotypic stasis in this complex (assuming that phenotypic convergence is not at play here). In order to test for the role of natural selection driving phenotypic stasis, observational and/or experimental data is required to measure the fitness differentials and trait heritabilities [274]. Moreover, a range of evolutionary processes might equally explain the pattern of phenotypic stasis, including stabilizing/fluctuating selection or low evolutionary rates [275], and genetic constraints [276]. Grummer and colleagues [238] revisited the phylogeny of the *L. fitzingerii* species group using genomic sequence-capture data and found a pattern of recent and rapid speciation, unresolved relationships and reticulations within this clade. This lack of resolution has been problematic in several phylogenetic studies of *Liolaemus*, which have frequently found polytomies within the genus using multi-locus data sets [277,278,279]. However, these datasets were small relative to the number of loci needed to distinguish between hard versus soft polytomies under some speciation scenarios. Thus, these clades are excellent candidates for follow-up studies implementing new analytical approaches developed to test for rapid radiations ([280,281], but see [282]). At a macroevolutionary level, Olave et al. [283] used an explicit model in a statistical coalescent framework to test for rapid radiations in *Liolaemus*, in a sample of 142 species of the subgenus *Eulaemus*. They used datasets simulated under explicit evolutionary models (including rapid radiations), and tested them against empirical data [283]. They found support for two rapid radiations as the most plausible hypothesis for the diversification of *Eulaemus*. Studies that are more recent have revealed that these extremely species-rich radiations have been associated with shifts in the diversification rates [284], and the adaptive processes linked to an episodic ecological opportunity generated by the gradual uplift of the Andes [285,286].

Clarifying the real species diversity of *Liolaemus* may be complicated by several factors. First, fuzzy resolution of species limits may be due to the small sample sizes (only 1–3 individuals for some localities), especially when using methods for which a minimum of five is recommended [287]. Further, some species are known only from their type localities, which may compromise the collection of sufficient bioclimatic, morphological, and/or molecular data.

Species delimitation in most species’ complexes of *Liolaemus* has also been complicated by the occurrence of extensive paraphyly in multiple clades. This paraphyly results in an incongruence between mtDNA and traditional morphological species limits. Some of these cases are due to incomplete taxonomic knowledge [288,289]. For others, incomplete lineage sorting [288] and hybridization were suggested as the most likely causes [271,272,277,290]. In some cases, further study based on nuclear markers has confirmed mtDNA introgression [58,291], More in-depth assessments with multiple loci and novel coalescent-based methods in the *L. boulengeri* and *L. rothi* complexes [292] and the *L. fitzingerii* group [293], further suggest that hybridization has played a major role in *Liolaemus* diversification. The incorporation of genome-wide markers should help tease apart the relative contributions of lineage sorting versus introgression in *Liolaemus*. However, recent simulations suggest that massive genomic data could bias species delimitation methods to detect interspecific divergence even when pervasive gene flow between lineages is more consistent with intraspecific structuring [294]. Given the reality of gray zones in speciation processes and the increased resolution of divergence patterns based on genomic data, delineating species boundaries in some cases may never be straightforward [295].

An appropriate understanding of macroevolutionary patterns depends on the clarification of the actual diversity of the genus and of the species boundaries within several complexes. Incomplete taxon sampling might bias inferences of macroevolutionary patterns, so correction methods may need to be employed [296]. For instance, the lack of complete sampling in most phylogenetic studies of *Liolaemus* might have also biased branch-length estimates due to the node density artifact [297]. If this artifact is actually present (e.g., based on the ‘delta’ statistic of Webster et al. [298]), the implementation of phylogenetic mixed models might help to solve, or at least alleviate the problem that could bias divergence time estimates [299]. The increased lineage sampling appears even more important given that distinct patterns of diversification and trait evolution have been found in different clades of *Liolaemus* [284]. Moreover, Olave et al. [284], found that the high diversification rates in *Liolaemus* seems to be actually a result of lower extinction rates, relative to its sister genus, *Phymaturus*. In addition to a better knowledge of α-taxonomy of the genus, it is also necessary to obtain a well-resolved and robust phylogeny for the genus. This endeavor is proving difficult, despite the incorporation of genomic-level data [283,293]. This may be a consequence of the rapid diversification during the early and recent evolutionary history of several *Liolaemus* species complexes [283,293].

In cases where parapatry and introgression are limited or absent, some hypotheses of the drivers of speciation can be formulated based on comparing patterns of the variation in multiple data sets collected from recently diverged sister clades [43]. Given the current progress in our knowledge of the taxonomy and distribution of this genus, and the increasing availability of multiple data types, advanced studies of speciation processes in *Liolaemus* are able to be undertaken. For example, molecular, morphological, and niche envelope data for *L. petrophilus* have suggested that environmental niche divergence may have promoted diversification in allopatry, for sister clades north and south of the Somuncurá Plateau in Argentina [300]. It is suggested that the additional study of color, color patterns, and behavior in combination with previous datasets might shed light on the potential role of social signaling traits [301] in speciation in *Liolaemus*. In relatively closely related iguanian lizard families, these processes have been demonstrated to drive population divergence and a process of socially-mediated speciation (e.g., *Uta stansburiana* [183]; *Ctenophorus* [301]).

The discovery of parapatric hybrid zones in *Liolaemus* [277,288,290,302] is expected to prompt studies that are geared towards elucidating other evolutionary forces that could be involved in the origin and maintenance of this clade’s diversity. Considering that introgression and hybridization are common among species of *Liolaemus*, an accurate estimate of the phylogeny of the genus should take into account evolutionary reticulation processes using species network approaches (see Section 2.5). The best option may be the application of these methods to well-supported clades within *Liolaemus*. In addition, a new isolation-with-migration demographic model that relaxes the assumption of a fixed species tree (IMa3, [303]) looks promising for the study of speciation processes among closely related species that have diverged with the gene flow/introgression. Furthermore, admixture models that consider discrete migration restricted to specific periods can also be evaluated with new composite-likelihood, genome-wide approaches [144].

Another interesting research venue is the high degree of hybridization within *Liolaemus* [304]. For instance, a question arises whether this apparent morphological stasis is an adaptive feature in *Liolaemus*, at least partially maintained by recurrent hybridization, that may be associated with lower extinction rates in comparison with the more specialized sister genus *Phymaturus* [284]. For example, Olave et al. [273] have found evidence of morphological stasis driven by selective pressures in the *L. rothi* species complex, which probably reflects a common pattern in other *Liolaemus* complexes as suggested by previous studies (e.g., *L. kriegi* [305] or *L. bibronii* [306]). Based on these studies, a more dynamic evolutionary view of the lizard genus *Liolaemus* is emerging. This promises to offer many future opportunities to address how this very-species rich lizard genus has rapidly-diversified across the Andean/Patagonian landscapes of southern South America.

### 3.2. Lizard Speciation across the South American Dry Biomes

**Figure 1:**
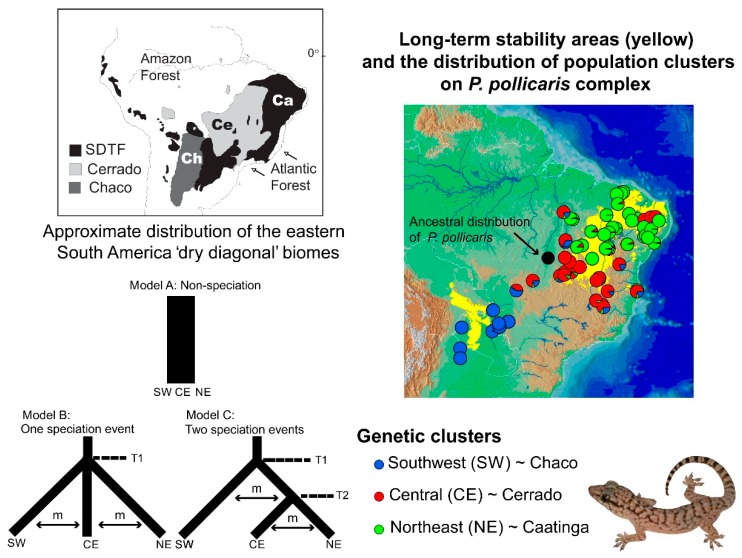
The distribution of the eastern South America dry diagonal biomes (top left) and the distribution of inferred Bayesian population clusters and ancestral distribution of *P. pollicaris* with respect to the inferred historical stability surface in yellow (stable areas obtained by overlapping predicted logistic outputs under four climatic scenarios: Current, 6, 21, and 120 kyr BP) and a digital elevation model for South America (brown represents higher altitudes). The pie charts represent the posterior probability that a given individual is assigned to a particular cluster. Alternative divergence models tested using an approximate Bayesian computation (ABC) framework (bottom left). STDF—Seasonal Tropical Dry Forest, T1—early divergence event, T2—recent divergence event, m—empirical relative mutation rates. Adapted from Werneck et al. (2012 [310]).

Speciation research has a great potential to reveal the contrasting roles of the geological landscape and changing climate on the diversification of amphibians and reptiles (and other groups), both indirectly (on small geographic scales [307]) and explicitly (over wider geographical scales [308,309,310]). Phylogeographic studies of speciation at multiple spatial and temporal scales can help elucidate the origins of biogeographic patterns. However, their ability to elucidate these processes depends on the geographic sampling, the biology of the taxa studied, and the nature of the markers used. Some empirical studies have integrated dense sampling with model-based parameter estimation and hypothesis testing for species delimitation [291]. However, the integration of model-based approaches with explicit historical biogeographic hypothesis for the Neotropical herpetofauna is still less explored. This integrative approach was used recently to study lizard speciation across the South American diagonal of dry biomes. The highly threatened open vegetation biomes of central-eastern South America extend diagonally across a large latitudinal range (Figure 1). They include the seasonally dry tropical forests (with the largest area, Caatinga, in northeastern Brazil), the Cerrado Savanna (central Brazil), and the Chaco (southwestern South America). Early studies have suggested an impoverished fauna (compared to the tropical rainforests), but these biomes are now recognized as having high diversity and endemism levels for amphibians and reptiles [311,312,313], as well as other taxonomic groups.

Recent studies in this region have advanced society’s understanding of the biogeographical processes responsible for speciation patterns in the amphibians and reptiles there. These studies have revealed some congruent patterns. The first commonality is the occurrence of genetic breaks geographically congruent with the limits of the three biomes. These breaks indicate complex speciation scenarios that may have been influenced by altitudinal variation [310,314]. Second, some geomorphological components have important roles in speciation, such as the Serra Geral de Goiás, the Serra do Espinhaço and the São Francisco River [310,314,315]. Third, deep divergences exist within closely related groups dating to the Miocene-Pliocene transition. These splits may be related to events such as the uplift of the Brazilian Shield and to marine introgressions [310,316,317,318,319]. Fourth, Cerrado lineages appear to have a deeper genetic structure when compared to Caatinga lineages. These Caatinga lineages have more shallow genetic structures, possibly indicating instances of ecological speciation, speciation with the gene flow [239,310], or recent demographic expansion [315,320]. Fifth, the prevalence of deep phylogeographic structures with high levels of cryptic diversity [310,314,316,318,319]. Sixth, the existence of a west-east diversification pattern, especially in the Cerrado [310,314,316,318,319]. For *Vanzosaura* lizards, the pattern of east-west divergence is congruent with morphological variation, and a new taxonomic arrangement was proposed for the genus with the description of a new species endemic to the Cerrado [314].

Alternatively, other patterns do not show overall agreement between studies. These include the role of Pleistocene climatic and vegetational cycles on the population structure, and the correlation between areas of climate stability and high genetic diversity. For example, Pleistocene climatic cycles were shown to be important for the diversification of Cerrado treefrogs [316]. On the contrary, other studies explicitly tested the prediction that areas of long-term stability during Quaternary climatic fluctuations would have greater genetic diversity and corresponding phylogeographic structure, but did not find such an effect in the lizard species investigated [310,318]. Thus, the responses to Pleistocene climate fluctuations seem highly variable among taxa. Furthermore, the different evolutionary responses to changing climates other than population extinctions and range shifts may be more common than previously thought. The persistence in situ can occur, if the changing climate remains within the species’ physiological tolerance limits [321] and if the preferred habit persists. This seems to have occurred in the case of the rock-outcrop specialist gecko *Phyllopezus pollicaris* [310]. In these cases, phylogeographic signatures are expected to reflect events that have not been overwritten by Pleistocene climate dynamics.

Thus, stability is not an exclusive force in generating diversity (species and genetic) patterns. Moreover, climate change should not be unconditionally associated with the loss of diversity (i.e., extinction) without a critical evaluation of each biological system’s idiosyncrasies. The stability-instability dynamic is crucial to promote speciation along the dry diagonal. The patterns of persistence and/or susceptibility to climatic change may provide important insights about the responses to future environmental changes and long-term population viability. The long-term population viability is critical for establishing efficient conservation strategies. However, some taxa associated with the dry diagonal may be more susceptible than others to range oscillations and extinction from anthropogenic climate change. The allocation of conservation resources may be more effective if comparative studies can provide evolutionary histories of a diverse array of co-distributed dry diagonal endemics.

On the population level, Werneck and colleagues [310] used model-based approximate Bayesian computation (ABC) to test alternative population-divergence hypotheses for the *P. pollicaris* gecko complex. These hypotheses correspond to hypotheses of historical biogeography at the landscape level, for the South American dry biomes. Three hypotheses were outlined for this species, each incorporating the different population structures, divergence times, and the patterns of the gene flow between the populations in the three biomes (Cerrado, Chaco and the Seasonally Dry Tropical Forests/Caatinga). The first hypothesis was a null model of no speciation, reflecting the early views in the literature that species in the dry diagonal biomes would share a single evolutionary history. As a first alternative hypothesis, a speciation model was proposed that predicted one ancient divergence event in three major phylogeographic clades (southwest/Chaco, central/Cerrado and northeast/Caatinga). This hypothesis represents a speciation scenario triggered by older geological events. The second alternative hypothesis was a speciation model with two divergence events. First, an initial separation between the populations from southwest/Chaco and all the others, followed by a more recent ecological divergence event between central/Cerrado and northeast/Caatinga populations (Figure 1). Stronger support was found for the model with two divergence events (one considered allopatric speciation and the other ecological speciation) among lineages associated with the Chaco, Cerrado, and Caatinga. These results revealed a complex scenario of diversification among the dry diagonal biomes.

Oliveira et al. [239] used ABC to test four alternative diversification scenarios for a whiptail lizard (*Cnemidophorus ocellifer*) in the Caatinga. These scenarios included varying the divergence times, the migration estimates, and the demographic histories. The authors found support for speciation with the gene flow along an environmental gradient.

In summary, new studies are revealing insights into the diversity, biogeography, and diversification of the lizard fauna of the dry diagonal biomes. These studies show that while the transition zones between the three biomes may interrupt the gene flow and promote reproductive isolation, additional factors are operating within each biome. For example, ecological speciation may be particularly important in the Caatinga biome (Figure 1).

### 3.3. An Early Stage of Adaptive Ecological Speciation in European Fire Salamanders.

In this section, recent research on a population of fire salamanders (*Salamandra salamandra*) in western Germany is described, where individuals appear to be undergoing the early stages of ecological speciation, associated with different larval habitats. This section begins by describing the general phylogeography of the species in Europe, followed by the details of the diverging population near Bonn.

**Figure 2:**
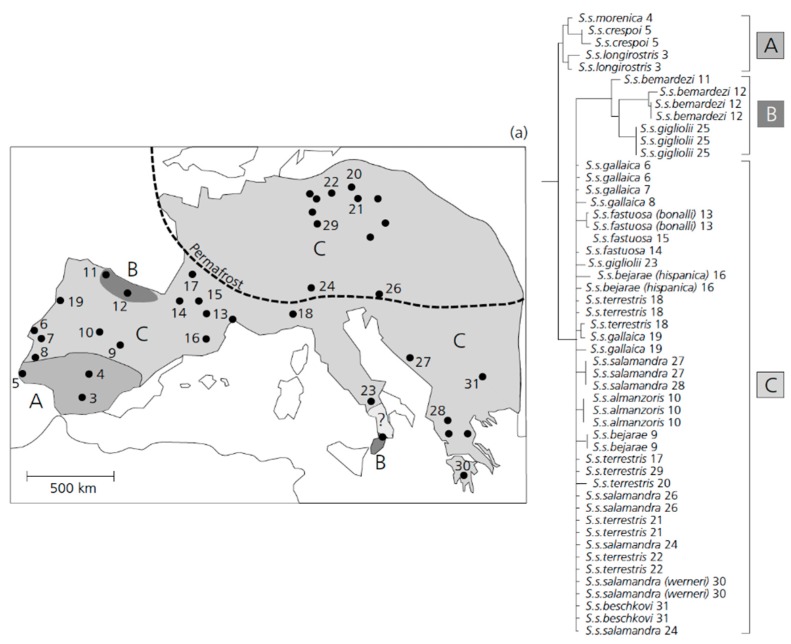
The geographic distribution of different clades within *Salamandra salamandra* across Europe derived from a population-based phylogeny of the mitochondrial D-loop [322]. The different shades of grey—the distribution of different phylogenetic clades. The distribution range of the subspecies *S. s. gigliolii* is uncertain (question mark). The dashed line—the approximate line of permafrost during the height of the last glaciation. Note that clade B shows an interrupted pattern by populations of clade C. (Modified after [322]).

Phylogeographic patterns observed between distinct fire salamander species were found to be quite different. The differentiation in the mitochondrial D-loop marker between populations of *S. salamandra* across Europe were found to be relatively shallow, especially when compared to Near Eastern fire salamanders (*S. infraimmaculata* [322]). The haplotypes of the mitochondrial D-loop of *S. salamandra* could be arranged into distinct clades occupying separate geographic ranges. The C-clade is distributed continuously across major parts of Europe except in southern Spain (Figure 2). Its existence is now verified based on both nuclear and mitochondrial genes [323]. Based on the observed population structure, members of the C-clade have colonized major parts of Central Europe (including all of Germany) following the last glaciation. This colonization followed the recolonization by native beech trees (*Fagus* sp.), which make up the natural forest habitats of *S. salamandra* [324] roughly 8000–9000 years ago. Therefore, these salamander populations must have become re-established quite recently in Middle Europe. Given this pattern, these salamanders provide an excellent system to study the consequences of habitat adaptation and lineage diversification in the recent past. In the ecological speciation framework described in Section 2, the incomplete instances of diverging populations can be subdivided into different stages. Here, the evidence that salamander populations in Germany correspond to an early stage (phase 1 or phase 2) of speciation is described. This system may be comparable to the well-studied three-spined sticklebacks in western Canada (see Section 2.3).

Fire salamanders in Central Europe typically deposit larvae in small permanent streams, in which they undergo development until metamorphosis is completed [325]. In the so-called Ville, an area composed of old broadleaf deciduous forests spanning from Cologne to Bonn, several large fire salamander populations can be found. Besides streams, some populations also use ephemeral habitats (e.g., small ponds, tire ruts, ditches) as larval deposition sites. As the risk of desiccation is high and the food supply is relatively low compared to streams, larvae developing in ephemeral aquatic habitats display several habitat-specific adaptations that are absent in stream larvae. These include a greater larval weight at birth, the ability to thrive on lower quality food sources, and early metamorphosis to escape unfavorable and non-predictable conditions [326,327,328]. Based on a detailed phylogeographic analysis of mt D-loop haplotypes across Germany [326], the Ville region was found to have been colonized by the western lineage of *S. salamandra* following the last glaciation. Since stream-reproduction is the ancestral condition, it can be hypothesized that pond-reproduction evolved locally in the range of the Ville after recolonization, no more than 8000–9000 years ago [326,329].

An extensive study of microsatellite loci showed that individuals were genetically differentiated in association with the two different larval habitats [329]. This study was conducted in the Kottenforst, an uplifted forest plateau in the Ville. The genetic differentiation might have been established under possible contact situations (i.e., in sympatry or parapatry) between stream and pond-adapted salamander types, as the dispersal rates have shown to be unexpectedly high in populations within the same range [330,331]. Accordingly, under a scenario of early adaptive/ecological speciation, assortative mating between differentially adapted ecotypes (pond versus stream) should underlie the observed genetic differentiation. Although clear evidence for assortative mating is missing, indirect evidence suggests that females show mating preferences under fully natural conditions. By reconstructing paternal genotypes from collected female offspring arrays, it could be shown that females preferred males that were more genetically similar to each other than expected by chance [332]. It therefore appears that females are able to discriminate between different males and do so under natural conditions.

**Figure 3:**
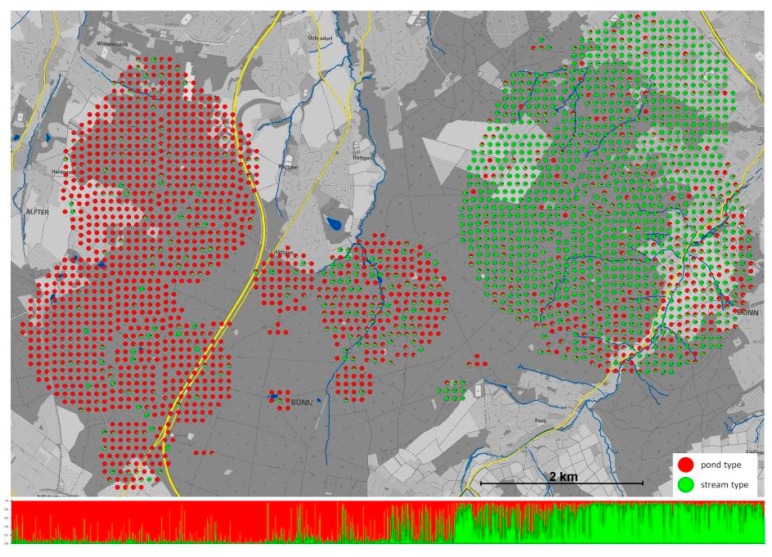
The adaptive divergence of the Kottenforst fire salamander population according to pond- and stream larval habitat. The fine-scale spatial distribution of 2653 genotypes representing individual salamander larvae sampled from pond and stream habitats across the Kottenforst. Each dot represents a single individual displaying as a pie chart the percentage assignment assuming two genetic clusters (K = 2). The bar plot composed of individual genotypes (each line represents a single larva) shows the corresponding assignment as represented by the pie charts from west to east across the Kottenforst. (From Hendrix et al. [334]).

It is difficult to predict whether the speciation process will continue or whether introgression will halt the divergence process at the present stage. Nevertheless, the adaptation to different larval habitats resulted in changes in many important traits. As expected, larval deposition behavior and maternal investment differs between pond- and stream adapted salamanders. The pond-type females extend larval deposition over an increased period and tend to deposit eggs more frequently compared with stream-type females [333]. Moreover, over successive deposition events, the body condition of larvae deposited by stream-type females decreased faster than larvae deposited by pond-type females. These differences in larval deposition behavior may represent a bet-hedging strategy, given that ponds are more likely to dry up than streams, and have more limited food availability. The prolonged deposition period might allow pond-type females to deposit larger larvae towards the end of the deposition period. Another important trait that differed between ecotypes is movement behavior and the dispersal of adult salamanders [334]. An integrative study was performed that combined passive integrated transponder tags (PIT tags) and radio transmitters with individual genotype-based habitat assignment of adults. This study showed that movement characteristics differed between the two ecotypes. The pond-adapted salamanders moved up to almost 2 km within two years of observation and displayed a typical distribution of long-distance dispersal among individuals. In contrast, stream adapted salamanders behaved in a manner consistent with short distance dispersal. Moreover, occupied home ranges of pond-adapted salamanders were considerably larger than stream-adapted ones. Overall, the higher movement flexibility of the pond-ecotype fits well with their unstable and less predictable larval habitat (Figure 3). It could therefore be shown that adaptation with the gene flow into different larval habitat types drives genetic divergence.

In addition, fire salamanders represent a promising system to address the genes and mechanisms enabling habitat adaptation. The development of species-specific microarrays allowed for the analysis of gene expression in different contexts [335]. Based on these results, parallel habitat adaptation and acclimatization of larvae in distinct fire salamander species (*Salamandra salamandra* versus *Salamandra infraimmaculata*) relies on the expression of different genes with a converging functionality [336]. A combined field and common environment study in the Kottenforst helped unravel the mechanisms underlying larval habitat adaptation to different microhabitats, such as water temperature regimes in each habitat. From 11,797 probes represented on the microarray-chip, 2800 genes were differentially expressed between the pond and stream larvae. Disentangling the effects of transcriptional plasticity from the genetic (evolutionary) divergence on the adaptation to the temperature revealed that 28% of the variance in the gene expression in nature could be attributed to plasticity and only a small fraction was affected by the genotype [337]. These results support a possible role of phenotypic plasticity in the diversification process.

In summary, fire salamanders offer a remarkable system to study adaptation to different habitats in the context of ecological speciation. Future research should address whether habitat-dependent assortative mating exists and how it is realized. Caudate genomes can be very large and therefore hard to sequence. The availability of a reference genome (e.g., [338]) would open new avenues to unravel the genetic basis of the changing traits in the context of the adaptation to habitat conditions further.

### 3.4. Body Size and Speciation Rates in Mantellid and Other Frogs

Speciation rates may be influenced by characteristics of the external environment and by intrinsic constraints from the organisms themselves. One well-studied intrinsic factor is body size, as discussed in Section 2. This case study of Malagasy frogs elucidates the role that body size has played in the speciation of the frogs of Madagascar.

**Figure 4:**
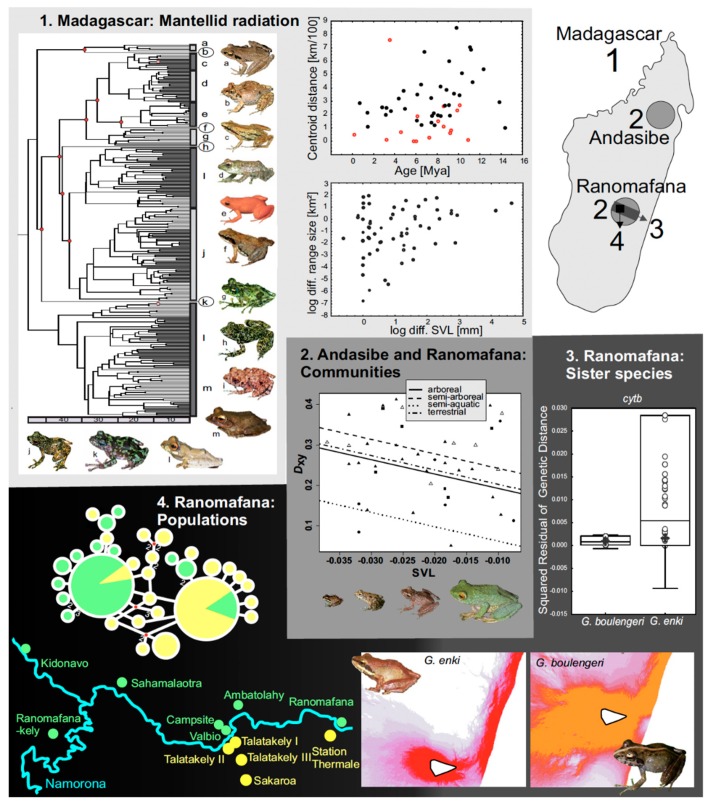
The importance of body size on amphibian diversification, from radiation to population. In Madagascar, the influence of body size on patterns and processes of evolution has been studied on several levels of the radiation, including (1) the complete radiation of mantellid frogs. Genera are abbreviated as follows: a, *Aglyptodactylus*; b, *Laliostoma*; c, *Blommersia*; d, *Guibemantis*; e, *Mantella*; f, *Wakea*; g, *Spinomantis*; h, *Boehmantis*; i, *Gephyromantis*; j, *Mantidactylus*; k, *Tsingymantis*; l, *Boophis.* SVL_ - Snout-vent length (2) The community level, comparing communities between sites of high diversity, Andasibe and Ranomafana, (3) A pair of mantellid sister species and (4) populations of one of these species. (1) Mantellid frogs of Madagascar constitute a species-rich amphibian radiation with high diversity of ecology and phenotype (tree). Young pairs of sister species are found in closer spatial proximity than older sister species pairs (top scatterplot), and sister species with different range sizes also differ in their body sizes (bottom scatterplot). (2) Mantellid divergence between sister species of two spatially separated communities is higher for smaller species indicating their more limited ability to disperse. (3) In a pair of ecologically similar mantellid sister species, *Gephyromantis enki* (smaller) and *G. boulengeri* (larger), the smaller species shows higher residual genetic variance across the same landscape than the larger species (box plot). Landscape resistance is lower for the larger species (inset maps; strength of landscape resistance is ranging from low—orange to high—red). (4) The population diversification for the small *G. enki* is influenced by barriers to dispersal such as the Namorona River (blue line) where localities on opposite sides of the river (yellow/green dots) are separated by a mutation in cytochrome b (indicated by the haplotype network with localities in corresponding colors). Figure references: Wollenberg et al., 2011 [34]; Pabijan et al., 2012 [348]; Wollenberg Valero, 2015 [199].

The endemic Malagasy frog radiations are well-known examples of adaptive radiation. They have been extensively studied for their phylogenetic relationships and biogeographic histories [6,33,34,339]. However, little is known about their ecologies, beyond general aspects such as habitat and breeding biology [340]. These frogs share Madagascar with other endemic clades (e.g., lemurs, tenrecs, Vanga birds), and the island is subdivided into several regions of biological turnover (another term for β diversity [201,341,342]). Thus, Madagascar offers a good model system to infer the processes causing species diversity, species richness, and endemism [201,343]. Most research in Madagascan frogs has been conducted on extrinsic factors, owing to the collection of large datasets on genetics and species distributions. The general finding for the entire radiation of Madagascan mantellid frogs was that many sister species occurred in close spatial proximity to each other [34] (Figure 4), and most species had very small ranges. Based on these findings, allopatric speciation across large distances was considered an unlikely mechanism for speciation. Furthermore, no evidence was found for a prevalence of dissimilar range sizes between sister species [34]. Thus, the results did not support the idea of peripatric speciation, which is speciation through isolation of peripheral populations [105]. Wollenberg et al. [34] found that clades of smaller species tended to have higher species diversity, smaller mean range sizes, and higher mitochondrial substitution rates. However, a small number of Madagascan frog species with large ranges were available, so the hypothesis of the body size correlated with the range size and the substitution rates needs further testing in other radiations that contain a diversity of body sizes and range sizes. Nevertheless, it can be concluded that these results are consistent with other recent studies showing a connection between the body size and lineage diversification [132,134]. In contrast, a small body size can alternatively be proposed to limit the number of dispersal events leading ultimately to speciation, so that a putative optimally speciating phenotype may in fact be of intermediate size (see below). A complication to infer such links between phenotype and speciation events in many mantellid species is their relatively old ages. In order to better link the pattern to the process, studies using phylogenetic comparative methods should optimally be supported by studies among populations.

Speciation is ultimately a consequence of processes occurring at the population level [344,345]. In this paragraph, the term speciation is used to refer to both the species formation and the origin of lineages within the species that may (or may not) complete the speciation process. As similar processes may drive the patterns of biodiversity both within and among species [346,347], a straightforward approach is to test whether factors affecting clade diversification might also affect genetic variability at the intraspecific level. Pabijan, Wollenberg and Vences [348] evaluated the contributions of five variables that might potentially influence speciation in frogs (body size, range size, reproductive mode, adult microhabitat and skin texture) on mitochondrial sequence variation in 40 species of rainforest frogs (Mantellidae) from Madagascar. Contrary to expectations, four out of five variables (range size, adult microhabitat preference, skin texture and reproductive mode) showed no relationship to (i) regional differentiation or (ii) levels of genetic variation within the populations (Figure 4). 

Nevertheless, body size was inversely correlated with nucleotide divergence between populations. The small-bodied and medium-sized frogs exhibited high F_ST_ values and an absence of haplotype sharing. This implies that substantial population subdivision is an outcome of low levels of gene flow in small-bodied mantellids and is corroborated by a lack of haplotype sharing in nuclear genes at least in some species [349]. On the other hand, most of the large species exhibited low genetic differentiation among the populations and evidence of haplotype sharing. Pabijan, Wollenberg and Vences [348] suggested that low dispersal ability most likely caused higher population differentiation in small-bodied mantellids. However, other mechanisms might have also contributed to this pattern (e.g., shorter generation times in small frogs or size-dependent metabolism determining mitochondrial mutation rate). Whatever the mechanism is, the lack of genetic cohesion among the populations establishes regional genetic isolation within mantellid species. This lack of cohesion may accelerate rates of speciation in smaller species. Some animals also show signatures of higher diversification in smaller-bodied lineages, but with clearly defined constraint values for very small body sizes [350], and the pattern is not evident across squamates [351] or animal phyla [127].

A consequence of higher regional genetic differentiation in small-bodied frogs might include increased speciation rates in clades containing small species. This hypothesis received no support from mantellids—small body size correlates with small range sizes and higher rates of nucleotide substitution, but not with increased rates of cladogenesis [34]. This apparent inconsistency between the microevolutionary process and the macroevolutionary pattern may stem from the cumulative influence that dispersal has on diversification at short and long temporal scales. In the long-term, small-bodied low dispersal species may have fewer opportunities to colonize suitable new habitats [134,352] which in amphibians could further be exacerbated by niche conservatism [83,96]. The range expansion in small-bodied species, facilitating allopatric speciation, would therefore be less likely to occur. Moreover, amphibians with geographically limited distributions might have higher extinction rates [353]. Thus, although small body size may potentially accelerate speciation via higher rates of nucleotide substitution and regional differentiation, the net diversification may be simultaneously offset by fewer chances for range expansion and higher extinction rates in poor dispersers.

Recent developments in dispersal theory have highlighted that speciation can occur at smaller spatial scales in taxa with low dispersal capacity [354], whereas high gene flow among populations usually inhibits speciation [355]. The highest species diversity (and presumably highest speciation rates) may occur in lineages with intermediate dispersal abilities that are sufficient to extend their geographic ranges, yet occur in low enough densities to maintain low levels of the gene flow, allowing for population differentiation [355,356,357,358]. As the range size and the range filling correlates with body size in amphibians (tested in Madagascar; [342]), it can be hypothesized that the intermediate dispersal ability corresponds to intermediate body size in frogs.

Evolutionary trends in body size have been repeatedly hypothesized to influence speciation and diversification in anurans. An evolutionary reduction in body size has often been accompanied by the truncation of the development of some morphological features (progenesis), such as skull elements and reductions in numbers and elements of the digits. The miniaturization in *Batrachoseps* (Plethodontidae) was thought to underlie fractal diversification (i.e., the non-adaptive radiation of morphologically and ecologically similar species through extreme range fragmentation [359]). A reduction of body size may have also initiated an ecomorphological radiation in the plethodontid genus *Thorius* [360]. However, a reduction of body size was not associated with the diversification rate in phrynobatrachid frogs [132]. On the other hand, large body size is part of a dispersal-prone phenotype and is linked to diversification in toads [134]. In general, body size is positively correlated with range size as recently shown in a comprehensive study of Malagasy amphibians and reptiles [342], reflecting higher dispersal capacity of large-sized animals. However, this association has not yet been analyzed in a large-scale macroecological study in amphibians. Likewise, no large-scale test of habitat associations and body size is available, even though many large-bodied temperate species (anurans and salamanders) seem to be associated with aquatic habitats, many large-bodied tropical species seem to be arboreal, whereas small species from both high and low latitudes seem to be more terrestrial [361].

Rodríguez et al. (2015 [362]) showed that both new world and old-world frog species living in non-forested lowland habitat showed low levels of a population structure. In contrast, the populations of rainforest species from mountainous areas were highly differentiated. The differences in dispersal ability were proposed to explain this result, with forest-adapted anurans thought to be less mobile than species dwelling in open areas. One pertinent corollary of these findings is that anurans from topographically complex rainforest areas (e.g., tropical mountains) should exhibit higher speciation rates assuming a predominance of allopatric speciation. This finding is in line with previous suggestions that heterogeneous topographies and mountainous areas may facilitate intraspecific divergence [363] and increase speciation or diversification rates [33] in frogs. For example, Hutter and colleagues [364] found accelerated rates of diversification in Andean frogs relative to those in other regions, such as the lowland Amazonian rainforest. In a study at a smaller spatial scale in Central American anurans, Paz and colleagues [365] identified body size, the reproductive mode, landscape resistance, geographic range, and biogeographic origin of lineages as the main predictors of phylogeographic patterns. This study highlighted species-specific life histories that may interact with landscape features and either promote or inhibit speciation, as also suggested in single taxon analyses [199,366].

Several other intrinsic species traits may be influencing speciation rates in amphibians, but are not known in sufficient detail in order to make firm conclusions. For instance, physiological and cellular processes affecting the DNA substitution rate may modulate the speciation rate in some amphibian lineages. The differences in active metabolic rates scale with substitution rates in both mitochondrial and nuclear genes in poison frogs [367], and clade level variation in metabolic rates may also contribute to patterns of substitution in mtDNA in salamanders [368]. If nucleotide substitution rates are positively correlated with speciation rates in amphibians, as they are in birds and reptiles [369], then it is anticipated that differences in metabolism among clades may also translate to different levels of species formation, although no influence of this trait was found on diversification rates across vertebrates [102]. Other potential but yet little-explored traits that may affect speciation rates include a variation in genome size [370] and karyotype instability [20,371,372].

### 3.5. Convergent Phenotypic and Genomic Adaptations to Elevational Clines in a Caribbean Anolis Species

The repeated evolution of similar adaptations to similar environments has been identified in several groups of amphibians and reptiles. For example, two different snake-like body forms evolved convergently within squamates [373]. Frogs from different clades converged on a limited number of ecomorphs associated with different microhabitats [374]. *Cryptoblepharus* lizards in Australia show a comparable pattern [375]. Pythons and boas have convergently evolved similar head shapes related to their ecological niche [376]. Convergent evolution among species is a particularly interesting outcome of the speciation process. If it appears in populations, it can also give insights into how extrinsic factors lead to similar phenotypes evolving in similar environments as genomes diverge.

One classic example of convergent evolution is the set of circa 140 species of Caribbean *Anolis* lizards. Among these species, there has been repeated evolution of similar sets of ecomorphs on different islands [377,378]. These ecomorphs are associated with different microhabitats, and they are characterized by distinct morphologies and behavior. They include the crown-giant, trunk-crown, trunk, trunk-ground, grass-bush, and twig types. Most of these ecomorphs have evolved convergently on each of the four largest islands of the Greater Antilles (Cuba, Hispaniola, Puerto Rico, and Jamaica [377]). Ecological speciation is a likely explanation for this in situ diversification of similar ecomorphs on different islands [377,378].

Despite the attention on the role of ecomorphs in driving this radiation, Caribbean *Anolis* lizards also include many younger speciation events that occurred within the same island and same ecomorph type (as evident from the high number of terminal taxa per island-branch and ecomorph in published phylogenies [378]). Some studies on *Anolis* have confirmed Ernest Williams’ [379] original hypothesis that diversification among younger species might be related to environmental differences (e.g., *Anolis cybotes* [380]). Additionally, some studies have found that sexual selection may be involved in divergence among populations (e.g., in *Anolis distichus* [381]). The different climatic regions on Caribbean islands harbor different species of anoles from the same clade and ecomorph category, and additional morphological variation that is associated with different macrohabitats is found within ecomorph categories (e.g., *A. cybotes* [380]; *Anolis roquet* [111,112]). Thus, Caribbean *Anolis* offer a model system to investigate convergent evolution among the populations, and to address whether the speciation process is deterministic or contingent. Contingency assumes evolution is strongly influenced by chance [382,383]. Determinism assumes that evolution occurs along more predictable trajectories [113,384,385,386,387]. Comparing the outcomes of speciation across different taxonomic and temporal scales in *Anolis* might provide insights on the common mechanisms of divergence [388].

*A. cybotes* is a trunk-ground ecomorph that is continually distributed across Hispaniola, which is both topographically and climatically heterogeneous [389]. *A. cybotes* show a strong genetic population structure and associated divergence in phenotypes. This phenotypic variation includes different perching habits on tree trunks and on rocks, keeled and unkeeled ventral scales, dewlap colors ranging from white to yellow and salmon-colored, and divergent skeletal measurements [380]. The morphological phenotypes are similar in high elevation populations in three different mountain chains (Sierra de Neiba, Sierra Bahoruco, and Cordillera Central). This has been shown through osteological measurements of over 500 specimens [36]. In these montane populations, *A. cybotes* have shorter limbs, wider skulls, and higher body mass, and occupy lower perches than in the lowlands. Based on a phylogeny among populations, this pattern indicates convergence [36]. Two of these populations, in the Cordillera Central and the Sierra Bahoruco, are currently placed in separate species (*A. shrevei* and *A. armouri*). Some authors even placed these high-altitude populations in a different genus (*Audantia*, first erected by Cochran 1934 [390], then later used for the entire cybotoid anoles clade [75]). However, *A. cybotes* populations were not clearly separated in a mitochondrial phylogeny [380]. Therefore, despite the three separate origins of convergent, montane phenotypes (and genetic divergence), a no-case for completed speciation at the genome level can be made yet.

**Figure 5:**
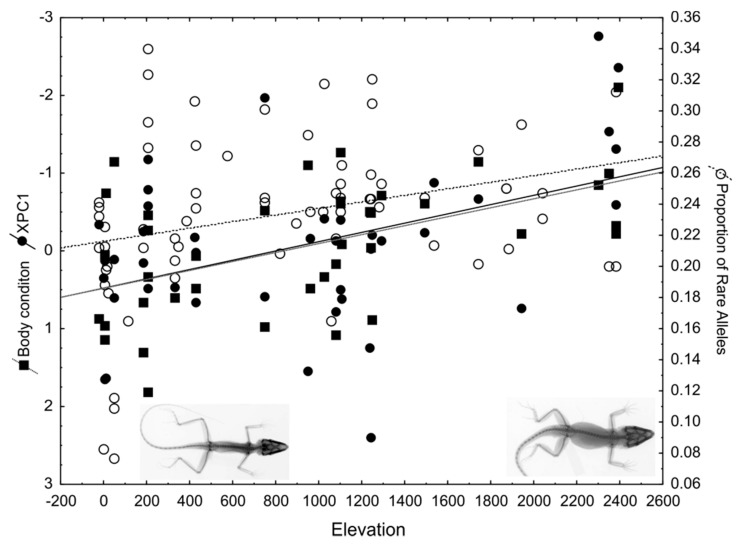
The covariation of rare allele frequencies of outlier RADseq SNPs with phenotypic adaptation to elevation (in meters above sea level). The transformed (residual to SVL and summarized via a principal component analysis) phenotypic variables (Wollenberg et al. [36]) representing body condition (relative body mass = snout-vent length/weight in g), and XPC1 (relative bone length, the variable shows shorter bones as larger values and is thus plotted inverse). The inset images show X-rays of typical lowland phenotypes of *A. cybotes* (left), and highland *A. cybotes* (right, own images). From Rodriguez et al. (2017) [391], under the Creative Commons license.

The adaptation to environmental gradients is a well-researched phenomenon (see Section 2, also [392,393]). However, finding multiple origins of convergent phenotypes within a single species is more surprising. Subsequently, several single nucleotide polymorphisms (SNPs) have been identified that differ between highland and lowland populations [391]. The frequency of rare alleles co-varies with elevation, together with osteology and relative body mass (Figure 5). Fourteen of these SNPs were located in genes with functions that have previously been linked to adaptation and to the temperature [391,394]. This pattern is consistent with an adaptive downshift in the lower critical temperature (CT_min_) at higher elevations [395], and that mirrors the global pattern of CT_min_ as a variable physiological trait [396]. Three of the 14 SNPs are found on one gene, CALCR (calcitonin receptor). This gene is known to regulate bone mineral density in humans [397,398], and is involved in preferred temperature selection and body temperature regulation across the animal kingdom [399]. These findings help to support the idea of an environmental factor, an elevation-related climate that independently selects for similar phenotypes based on genes with similar functions in different populations. Overall, these results support the idea of determinism.

According to Streelman and Danley [100], the diversification of lineages during a vertebrate adaptive radiation occurs in stages. The first stage encompasses divergence in habitats. The second stage encompasses divergence in trophic morphology, and the third stage divergence in communication. The aspects of divergence among *A. cybotes* populations could recapitulate the mechanisms of diversification into novel clades earlier during the Anolis radiation. Furthermore, finding congruence between completed diversification events and current or incomplete lineage diversification events could help to link the mechanism of speciation to patterns of speciation. Both *A. cybotes* populations, and the phylogenetic clade it belongs to (Hispaniolan trunk-ground anoles, containing *A. cybotes* and other species), had differences in the morphology associated with bioclimatic divergence [36], which corresponds to the first stage of diversification [100]. *A. cybotes* populations also had a small percentage of their morphology aligned to the occupation of different structural microhabitats (perches), which mirrored the mechanism of diversification among the Anolis ecomorphs that had occurred even longer ago. This evidence points at a strong signature of deterministic evolution [36]. However, another part of the overall morphological variance among *A. cybotes* populations was determined by a set of characters that were not observed previously to vary with divergence in anoles such as claw morphology, which can be interpreted as an element of contingency.

### 3.6. Galápagos Giant Tortoises: Dispersal, Allopatry and the Fusion-Fission Dynamics of Speciation

Oceanic island systems have provided valuable insights into the patterns and processes underlying speciation. Specifically, they can act as laboratories of evolution, with simplified and rapidly maturing biotas that aid in clarifying evolutionary processes that may be opaque in more mature ecosystems [400,401,402,403]. Moreover, many oceanic island systems provide replicated natural experiments and an explicit temporal component associated with the formation or separation of landmasses. The Galápagos Archipelago occupies a unique position in evolutionary biology. The islands have played a large role in influencing evolutionary theory from the time of Darwin, and have continued to be important for empirical evolutionary research to the present day [404,405]. The key question in this case study is whether speciation on island archipelagos is solely determined through allopatry, or which other mechanisms might be identified in such a classical setting for allopatric speciation.

**Figure 6:**
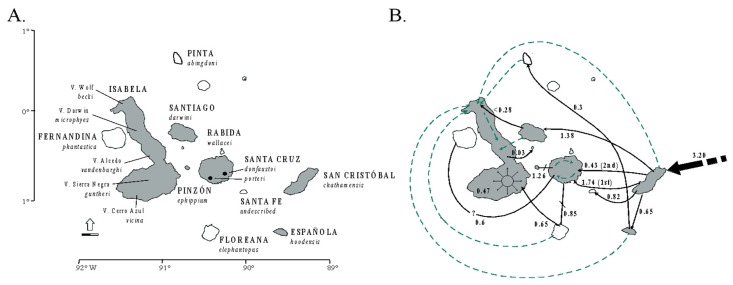
(**A**) The distribution of giant tortoises in the Galápagos Archipelago. The shaded and non-shaded islands indicate the presence of extant and extinct tortoise populations, respectively. The italicized names indicate current taxonomic designations. (**B**) A schematic of the proposed phylogeographic history of Galápagos giant tortoises modified from Poulakakis et al. (2012) [411]. The arrows represent dispersal and colonization events within Galápagos, with the numbers indicating approximate temporal order in millions of years. The short solid line segments indicate vicariance events. The solid black arrows are hypothesized natural colonization events, while the dashed arrows represent recent and likely human-induced translocations.

The islands are known to either select for small body sizes in larger animals, or for gigantism in species with smaller mainland relatives (called the Island Rule [406]). The Galápagos giant tortoises (*Chelonoidis* spp.) are such a group, for which evidence for a dispersal-and-vicariance mechanism in speciation is very strong. Their ancestors arrived in the islands from mainland South America approximately 6–12 million years ago (mya) [407,408] upon which the lineage diversified into 16 species of Galápagos giant tortoises. One of these was only recently described [409], and 5 others have previously gone extinct largely due to human activities (Figure 6). The key properties of this study system are longevity and long generation times in an island setting, which promises to offer insights into the speciation process and its resulting patterns in slow motion.

The diversification in Galápagos giant tortoises has long been considered to follow the progression rule (e.g., colonization sequences show a progression from older to younger islands; [257,403,410]). However, a recent study of all extant and extinct species paired phylogenetic analyses of mtDNA data and Bayesian inference of species divergence times, and combined them with paleogeographic reconstructions [411]. This study set forth both more complex hypotheses related to patterns of colonization, as well as timing and the mechanisms of divergence. The results implicated both allopatric isolation and dispersal as the mechanisms of diversification [411]. This study also provided critical information to guide conservation efforts [412].

Nevertheless, speciation is not always a bifurcating process. In some cases, it might be better represented through a complex network of fusions and fissions over time. Through their relatively unique life history traits, Galapagos giant tortoises may represent a good model system for understanding such evolutionary dynamics at the early stages of speciation [258], such as the impact of introgressive hybridization on speciation [413,414]. These processes may also lead to despeciation, as in the case of some Darwin’s finches [114].

In Galápagos giant tortoises, several introgressive events have been found, which seemingly led to very different evolutionary outcomes. A recent study on the population history of *Chelonoidis becki* endemic to northern Isabela Island has shown that two genetically distinct tortoise lineages independently colonized the slopes of Volcano Wolf on the island of Santiago [415]. Remarkably, these lineages appear likely to fuse back together after ~50,000 years of evolution in micro-allopatry [415]. This finding represents an unprecedented opportunity to look at the fusion/fission dynamics of early speciation, which are rarely captured in study systems with shorter generation times. Human translocations of giant tortoises are also likely responsible for rare introgression events between allopatric *Chelonoidis* species. Thus, humans have facilitated the dispersal across the archipelago. Early phylogenetic studies of extant Galápagos giant tortoise species noted rare the detection of aliens on Isabela and Santiago Islands, individuals with highly divergent haplotypes that were more closely related to those in geographically distinct populations from other islands rather than the local population [416]. These aliens were most abundant along the slopes of Volcano Wolf on northern Isabela Island. This is where non-native tortoises appear to have been deposited by whalers, a hypothesis consistent with old logbooks from the industry [258,410,416]. Subsequent studies that included population-level samplings of now extinct species (*C. elephantopus* from Floreana; *C. abingdonii* from Pinta) by way of historical DNA analysis of museum specimens confirmed the non-native origin of the Volcano Wolf aliens [417,418,419,420,421]. Given their rarity, the hybridization events may likely not affect the evolutionary trajectories of the tortoise species involved (e.g., *C. becki* lineages on Volcano Wolf [415]). Nevertheless, these events are of considerable conservation importance, as some hybrids contain genomic material from the now extinct species, such as those from Floreana (*C. elephantopus*) and Pinta Islands (*C. abingdonii*). The existence of highly divergent haplotypes is consistent with a reverse island syndrome, where island populations experiencing unpredictable environments with resulting fluctuating population sizes (e.g., by translocation to a new island) increases sexual selection [422]. A similar pattern has been found in invasive bullfrogs on small islands in China [423].

Recent and ongoing studies are devising strategies for using these hybrid individuals for the purposes of genetic rescue. This is part of a broader plan for reintroducing giant tortoises to islands where they have been presumed extinct [424,425]. Moreover, the publication of the Galapagos giant tortoise genome [426] and recent/on-going population genomic studies have enabled new and exciting opportunities to enhance society’s understanding of speciation within and among the islands. This includes a new understanding of the relative importance of introgression and fusion events in species formation and persistence, and the study of the genomic architecture of traits associated with their ecological and morphological diversification [427,428,429,430].

## 4. Conclusions and Outlook

Vences and Wake (2007) [431], the last published comprehensive review of amphibian speciation, pointed out that most general patterns of speciation are gained from studies of only a few well-known species, and discussed how intrinsic factors such as the reproductive mode and ecological specialization could direct the predominant mode of speciation and patterns of genetic diversity. At the time of that review, 5605 amphibians had been described. Since then, new conceptual advances, as well as novel technological developments in genetics and genomics [432], have since led to a subtle but important shift in perspective: Butlin et al. [124] argued five years later, that any categorization of general modes of speciation that predominantly apply to specific groups is ultimately unhelpful. Any categorization in the research of speciation only emphasizes aspects of the processes that ultimately work together in order to generate new species. As Vences and Wake had previously noted, different examples with partially contrasting evidence highlight the speculative nature of single-case correlations [431]. It is now known that speciation modes and mechanisms can be influenced both by intrinsic and extrinsic factors that operate together in the process of species formation.

**Figure 7:**
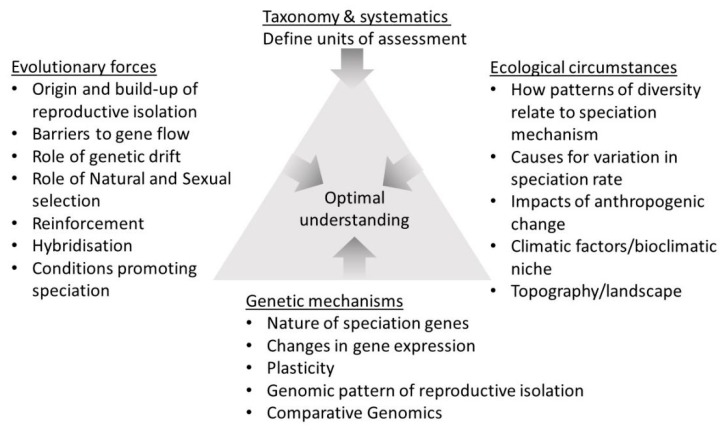
The different aspects contributing to speciation, modified after Butlin et al., 2012 [124].

Butlin and colleagues [124] argued that modern speciation research should instead be aligned to three areas of investigation: (i) The origin and build-up of reproductive barriers; (ii) the genetics of speciation; (iii) the patterns of species diversity. A deep understanding of speciation processes therefore requires that evidence be collected for all three aspects (Figure 7), and for many different species in order to identify generalities. As the authors reviewed in this contribution, a prerequisite to commencing this process is a good understanding of current taxonomy and systematics such that the units of evolution can be defined. Amphibians and reptiles are relatively well studied for taxonomy and systematics, as well as for their ecological circumstances. The genetic mechanisms contributing to macro and microevolution are increasingly inferred, and some evolutionary forces (e.g., selection, drift and gene flow) are often studied. In contrast, the origin and buildup of reproductive isolation (with the exception of some traits like color patterns) are still relatively unstudied, perhaps because laboratory selection studies are not common in reptiles and amphibians. In the future, clades of amphibians and reptiles that would be amenable to careful studies of the above three components [124] of modern speciation research should be targeted.

The authors are writing this review at a time where the 8000th species of amphibians have recently been described [433]. At the same time, the number of amphibian extinctions related to chytridiomycosis has reached 90 species, with over 500 species in decline [434]. Reptiles are also in widespread decline [435]. An interesting novel development in the study of amphibians and reptiles relates to urban speciation. For example, *Anolis* lizards can adapt to increasingly anthropomorphic environments [436,437]. There may be conditions under which adaptation to human-modified habitats can promote speciation [438]. At the same time, a better understanding of early speciation processes may clarify how anthropogenic climate change can shape the fate of populations [439]. In contrast, later stages of a clade’s evolution may explain the fate of their ancestors in relation to paleoclimatic changes [440]. Understanding the processes of past speciation is therefore a prerequisite to understanding and predicting processes operating at present. Hopefully, this review will result in a more profound understanding of speciation across a broader range of taxa and scenarios.
